# Endovascular treatment with Viabahn stent-grafts for arterial injury and bleeding at the visceral arteries: initial and midterm results

**DOI:** 10.1007/s11604-021-01192-8

**Published:** 2021-09-04

**Authors:** Tatsuo Ueda, Satoru Murata, Hiroyuki Tajima, Hidemasa Saito, Daisuke Yasui, Fumie Sugihara, Shohei Mizushima, Takahiko Mine, Hiroshi Kawamata, Hiromitsu Hayashi, Shin-Ichiro Kumita

**Affiliations:** 1 Department of Radiology, Nippon Medical School Hospital, 1-1-5 Sendagi, Bunkyo-ku, Tokyo 113-8603 Japan; 2 Center for Interventional Radiology, Teikyo University Chiba Medical Center, 3426-3 Anesaki, Ichihara, Chiba 299-0011 Japan; 3 Department of Diagnostic Radiology, Saitama Medical University International Medical Center, 1397-1, Yamane, Hidaka, Saitama 350-1298 Japan; 4 Department of Radiology, Nippon Medical School Chiba Hokusoh Hospital, 1715 Kamagari, Inzai, Chiba 270-1694 Japan; 5 Department of Radiology, Nippon Medical School Musashikosugi Hospital, 1-396 Kosugi-machi, Nakahara-ku, Kawasaki-shi, Kanagawa 211-8533 Japan

**Keywords:** Viabahn stent-graft, Arterial injury, Arterial bleeding, Visceral artery

## Abstract

**Purpose:**

The purpose of the study is to evaluate the initial and midterm efficacy and safety of endovascular treatment (EVT) using Viabahn stent-graft (SG) for arterial injury and bleeding (AIB) at the visceral arteries.

**Materials and methods:**

Consecutive patients with visceral AIB who underwent EVT using Viabahn between January 2017 and February 2021 were retrospectively reviewed. Technical success, clinical success, peripheral organ ischemia, peri-procedural complications, bleeding-related mortality, 30-day mortality, neck length, re-bleeding, endoleaks, and patency of the SGs at 1, 3, 6, and 12 months were evaluated.

**Results:**

EVT using Viabahn was performed in 14 patients (mean age: 68.6 years; 12 males) and 15 arteries. The technical and clinical success rates were 100%. The rates of peripheral organ ischemia, peri-procedural complications, bleeding-related mortality, and 30-day mortality were all 0%. The mean neck length was 9.9 mm. No endoleaks or re-bleeding occurred during the follow-up (mean: 732 days). The SG patency was confirmed after 1, 3, 6, and 12 months in 78.6%, 78.6%, 78.6%, and 56.1% of the patients, respectively.

**Conclusion:**

EVT using Viabahn for AIB at the visceral arteries was safe and effective. SG occlusions without ischemia often occurred after 12 months.

## Introduction

Arterial injuries and bleedings (AIBs), including pseudoaneurysms and extravasation are often caused by surgical procedures, endovascular procedures, trauma, or infection. Conventionally, AIBs have been treated surgically; however, currently, a less invasive endovascular approach is generally preferred. Trans-arterial embolization for AIBs with embolic materials, such as coils, plugs, gelatin sponge, polyvinyl alcohol, and N-butyl-cyanoacrylate, is an established endovascular treatment (EVT) [1–10]. However, trans-arterial embolization has a risk of peripheral organ ischemia owing to embolization of the parent vessels. Therefore, indications for trans-arterial embolization must be assessed carefully to avoid ischemic complications. EVT with stent-grafts (SGs) is an ideal treatment for AIBs because it simultaneously permits hemostasis and maintains the blood flow to peripheral organs [11–13].

Viabahn is a heparin-coated self-expandable SG for peripheral arteries and exhibits high flexibility and accuracy of the delivery system. In December 2016, Viabahn SG was approved for arterial injury use in Japan for the first time in the world. Compared with that of a balloon-expandable SG, this high flexibility seems to be more suitable for endovascular repair of tortuous arteries such as visceral arteries. Therefore, EVT with Viabahn SGs for AIBs may be one of the best treatment options and is suitable not only for limb artery injuries but also for visceral artery injuries. Nevertheless, to date, reports regarding the initial and midterm outcomes of EVT with Viabahn SGs for AIBs are limited [14–20].

Endoleaks (ELs) could occur following SG treatment, and type 1 ELs could be associated with re-bleeding following SG treatment. Neck length is one of the most important factors in avoiding type 1 ELs in SG treatment [21, 22]. Therefore, type 1 ELs associated with SG treatment for visceral arteries may be associated with an increased risk of re-bleeding following the procedure.

This study aimed to evaluate EVT using Viabahn SGs for AIB at visceral arteries, focusing on initial and midterm efficacy and safety. Additionally, the relationship between neck length and type 1 ELs was assessed.

## Materials and methods

### Patients

This study included consecutive patients who underwent emergency EVT using Viabahn SGs for AIBs between January 2017 and February 2021. The eligibility criteria for EVT with SGs were as follows: (1) evidence of AIBs at the visceral artery, including pseudoaneurysms or extravasation on computed tomography angiography (CTA), (2) diameter of the target vessel between 4 and 12 mm, and (3) no contraindications to heparinization and contrast media. According to the instructions for use of Viabahn SG, the device indication in vessel injury is confined to the arteries in the "body trunk" (except the aorta, coronary artery, brachiocephalic artery, carotid artery, vertebral artery, and pulmonary artery). Therefore, we have excluded the locations outside of those indicated in the instructions for use from this study. This study was approved by the Institutional Review Board of our hospital, and informed consent was obtained from all patients before treatment.

### Endovascular procedure

EVT was performed through a common femoral or brachial artery with a 4-Fr sheath (Supersheath; Medikit, Tokyo, Japan) under local anesthesia. A 4-Fr catheter (GLIDECATH; Terumo, Tokyo, Japan) was advanced to the distal side of the injured artery with the aid of a 0.035-inch guidewire (Radifocus Guide Wire M; Terumo, Tokyo, Japan). The guidewire was exchanged for a 0.035-inch stiff wire (Amplatz Super Stiff™; Boston Scientific, Natick, MA) to exchange the sheath for a 6-7-Fr guiding sheath (Destination; Terumo, Tokyo, Japan). The guiding sheath was then advanced to the distal side of the injured artery in most cases. In cases where it was dangerous or impossible to advance the guiding sheath to the distal site of bleeding points, a guiding sheath was placed as close as possible to the bleeding point followed by advancing the SG alone over the bleeding point. A 0.018-inch stiff wire (V-18™, Boston Scientific, Natick, MA) was used for SG delivery. The injured artery was measured by arteriography or pre-operative CTA imaging. The diameter of the SG was sized to approximately 110% of the diameter of the injured artery. The length of the SG was such that it covered the entire injured artery. The Viabahn (Gore, Flagstaff, AZ) SG (5–7 mm in diameter and 25 or 50 mm in length) was advanced until it covered the entire site of the injured artery, and was then deployed. In case there was a branch artery of > 2 mm in diameter within 5 mm of the injured artery, embolization of the branch artery was performed with coils (Tornade; Cook Medical, Bloomington, IN, Interlock; Boston Scientific, Natick, MA) or an AMPLATZER™ Vascular Plug 2 (AVP 2; St. Jude Medical, St. Paul, MN) to avoid a type 2 EL. An angiogram was immediately performed after the deployment without post-dilatation of the SG. In case the angiogram revealed a type 1 EL, an additional percutaneous transluminal angioplasty with the same SG diameter was performed to treat the EL. After the procedure, if there were no contraindications, anticoagulation therapy based on dual antiplatelet therapy (aspirin 100 mg/day and clopidogrel 75 mg/day) was prescribed for at least 6 months to prevent SG thrombosis. A follow-up CTA was performed at 1, 3, 6, and 12 months following treatment.

### Assessment of EVT efficacy

The following data were collected: enrolled patient number, case number, age, sex, cause, presence of biliary or pancreatic leakage, drainage for the leakage, shock index (the heart rate divided by systolic blood pressure), indication, location of artery, approach site, diameter of the injured artery, diameter of the SG, oversize percentage of the SG, length of the SG, neck length, associated embolization, additional percutaneous transluminal angioplasty, procedure time, anticoagulation therapy, and CTA follow-up duration. The neck length was defined as the minimum distance between the edge of the SG and the injured or bleeding point. The technical success, clinical success, ischemia of peripheral organs, peri-procedural complications, bleeding-related mortality, 30-day mortality, re-bleeding, ELs, and SG patency at 1, 3, 6, and 12 months were assessed. Technical success was defined as the disappearance of the pseudoaneurysm and extravasation with preserved blood flow of peripheral organs on the angiogram. Clinical success was defined as complete hemostasis within 30 days following the procedure. Ischemia of peripheral organs was defined as ischemic sequelae of the peripheral organs of the injured artery. As per the Society of Interventional Radiology classification, complications were defined as major complications that required therapy [23]. Re-bleeding, ELs, and SG patency were evaluated using CTA images.

### Statistical analysis

Continuous variables are presented as means ± standard deviations, whereas categorical data are presented as percentages. Kaplan–Meier analyses were performed for SG patency using SPSS software version 21 (IBM Corp., Armonk, NY). Factors that might have influenced the patency of the SG (age, sex, anticoagulation therapy, location, artery diameter, SG diameter, oversize SG, and infection) underwent univariate analysis using the log-rank test.

## Results

### Patient characteristics

**Fig. 1:**
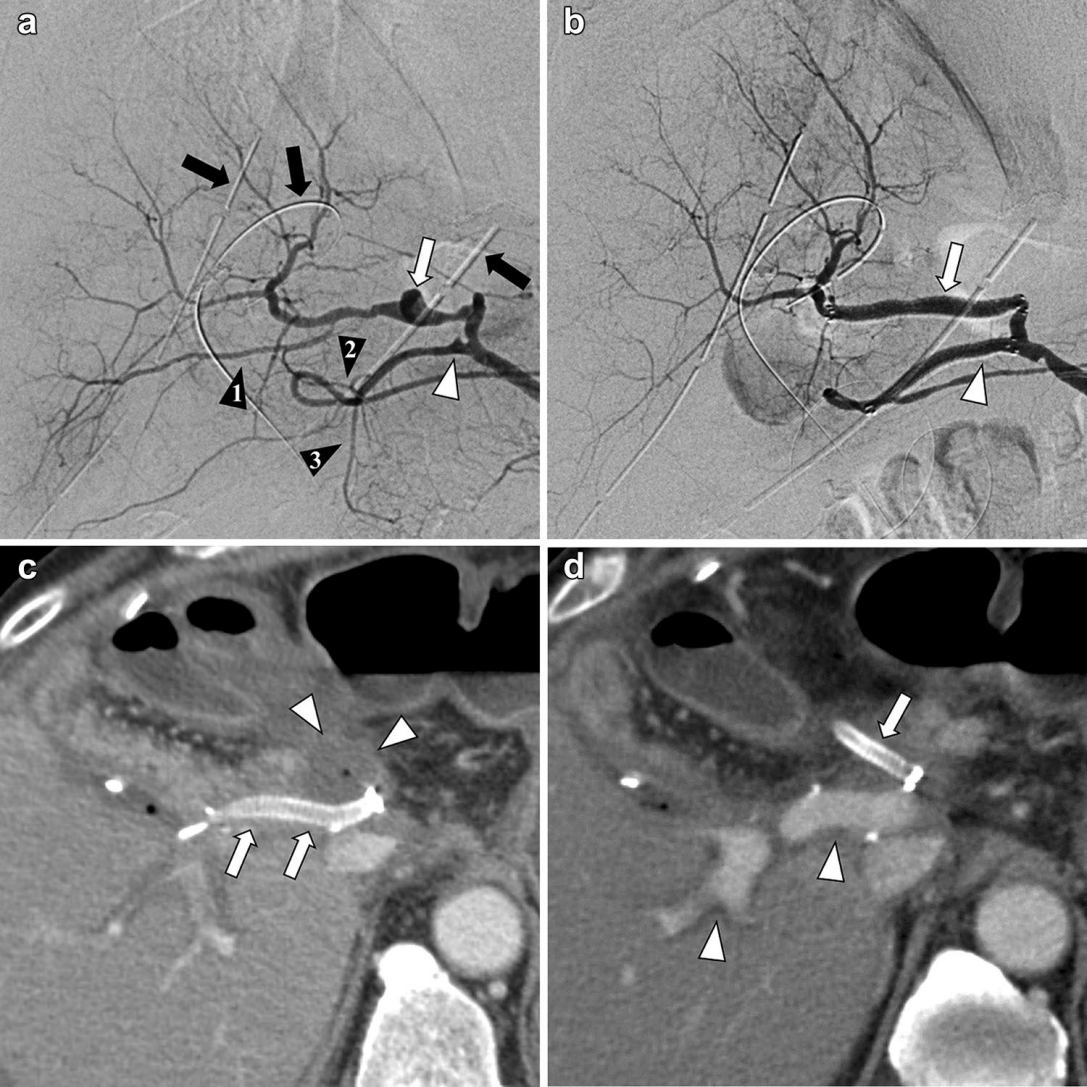
A 78-year-old man with post-left hepatic trisegmentectomy. **a** The pre-treatment angiogram shows two pseudoaneurysms in the right hepatic artery (RHA) (white arrow) and the gastroduodenal artery (GDA) (white arrowhead). The hepatic artery (A6) (black arrowhead 1) branches from RHA, and posterior superior pancreaticoduodenal artery (PSPDA) (black arrowhead 2) and anterior superior pancreaticoduodenal artery (ASPDA) (black arrowhead 3) branches from GDA. Three drainage tubes (black arrows) are placed in intraperitoneal space. **b** The angiogram after endovascular therapy with two Viabahn stent-grafts (RHA; 5 mm × 50 mm [white arrow], GDA; 5 mm × 50 mm [white arrowhead]) shows the disappearance of the pseudoaneurysms and a maintained blood flow of peripheral organs. The hepatic artery (A6), PSPDA, and ASPDA are occluded by Viabahn stent-grafts. There are no endoleaks on the angiogram. **c** Arterial phase of contrast-enhanced computed tomography (CECT) 7 days after treatment indicates the occlusion of the Viabahn stent-graft in RHA (white arrows). There is an abscess surrounding the stent-graft (white arrowheads). **d** Portal phase of CECT 7 days after treatment indicates the occlusion of the Viabahn stent-grafts in GDA (white arrow). Portal vein (white arrowheads) blood flow is maintained

The patient characteristics are presented in Table 1. The study included 14 patients with 15 AIBs. One patient had two simultaneously injured arteries (right hepatic artery [RHA] and gastroduodenal artery [GDA]) (Fig. 1a–c). The causes of AIBs were post-operative (*n* = 10), trauma (*n* = 2), acute pancreatitis (*n* = 1), and idiopathic (*n* = 1). The operations included subtotal stomach-preserving pancreatoduodenectomy (*n* = 4), pancreatoduodenectomy (*n* = 1), laparoscopic distal pancreatectomy (*n* = 1), laparoscopic pancreatoduodenectomy (*n* = 1), left hepatic trisegmentectomy (*n* = 1), extended left hepatectomy (*n* = 1), and gallbladder bed resection (*n* = 1). Among the 10 post-operative cases, 8 cases showed biliary or pancreatic leakage, and all of them underwent drainage for the leakage.

**Table 1:** Summary of results

Factor	
Patients	14
Cases	15
Age (years-old, mean ± SD)	68.6 ± 11.7 (range 50–84)
Sex (male/female)	12/2
Cause (post-op/trauma/pancreatitis/idiopathic)	10/2/1/1
Shock index (mean ± SD)	0.8 ± 0.3 (range 0.5–1.3)
Indication (pseudoaneurysm/extravasation)	12/3
Location of Artery (SA/CHA/PHA/RHA/GDA/CA/SMA)	4/4/3/1/1/1/1
Approach site (femoral/brachial)	13/2
Artery diameter (mm, mean ± SD)	4.7 ± 0.7 (range 4–6)
SG diameter (mm, mean ± SD)	5.5 ± 0.6 (range 5–7)
SG oversizing (%, mean ± SD)	18.0 ± 12.2 (range 0–40)
SG length (2.5/5 cm)	2/13
Neck length (mm, mean ± SD)	9.9 ± 4.9 (range 1–17)
Procedure time (min, mean ± SD)	43.4 ± 15.3 (range 15–66)
Technical success (%)	100
Clinical success (%)	100
Ischemia of peripheral organs (%)	0
Peri-procedural complications (%)	0
Bleeding related mortality (%)	0
30-days mortality (%)	0
Anticoagulation therapy (%)	78.6
CTA follow-up duration (days, mean ± SD)	731.7 ± 500.2 (range 39–1307)
Re-bleeding (%)	0
Endoleaks (%)	0
SG patency (%)	
1 month	78.6
3 months	78.6
6 months	78.6
12 months	56.1

*CA* celiac artery, *CHA* common hepatic artery, *CTA* computed tomography angiography, *GDA* gastroduodenal artery**,** *PHA* proper hepatic artery*, Post-Ope* post-operation, *RHA* right hepatic artery, *SA* splenic artery, *SD* standard deviation, *SG* stent-graft, *SMA* superior mesenteric artery

### Procedure results

The procedure results are presented in Table 1. The brachial artery approach was used for cases that exhibited a sharp angle downward between the celiac artery and aorta. Branch artery embolization was performed in 2 cases. Angiograms just following SG placement revealed type 1 ELs in 3 cases; however, all the ELs disappeared following an additional balloon percutaneous transluminal angioplasty and/or second SG placement. Consequently, there were no ELs on the final angiographies.

### Initial and midterm results

**Fig. 2:**
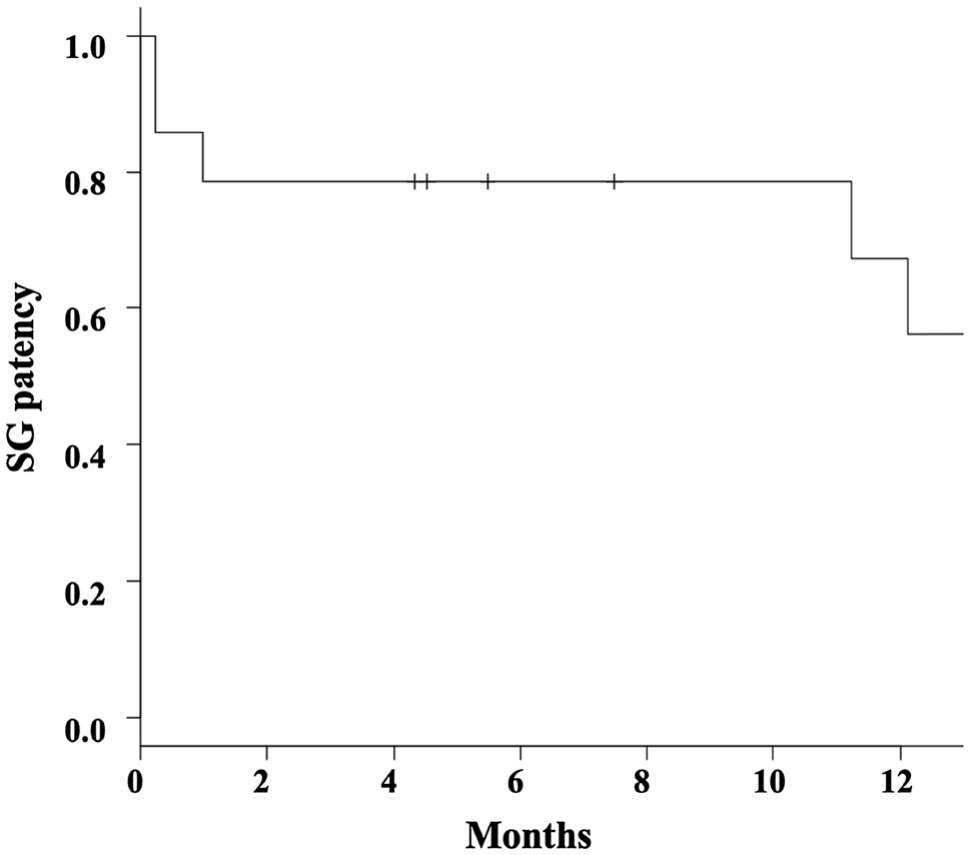
Stent-graft patency. A Kaplan–Meier curve reveals stent-graft patency overtime for Viabahn grafts. The transverse axis shows the time (months) after the procedure. *SG* stent-graft

The initial and midterm results are presented in Table 1. Technical and clinical success was obtained in all patients. Ischemia of peripheral organs, peri-procedural complications, bleeding-related mortality, and 30-day mortality were all 0%. Anticoagulation therapy based on dual platelet therapy was prescribed for 11 of 14 patients (78.6%), and 3 patients did not undergo this therapy because of contraindications with active bleeding. CTA post-operative follow-ups were performed for 14 of 15 patients (mean CTA follow-up periods: 731.7 days; range 39−1307). CTA imaging assessment of the SGs with information regarding AIB exclusion and SG patency was available after 1 month (*n* = 14 cases), 3 months (*n* = 13), 6 months (*n* = 10), and 12 months (*n* = 9). SG occlusion occurred in 5 cases (2 cases after 7 days, 1 case after 1 month, 2 cases after 12 months) within 12 months, and 1 case in 15 months. Among the 6 occluded SGs, the target artery diameters were 4 mm in 3 cases (3/6: three of six 4-mm diameter cases), 5 mm in 2 (2/7), and 6 mm in 2 (1/5). Furthermore, the SG diameters were 5 mm in 3 cases (3/8: three out of eight 5-mm diameter cases) and 6 mm in 3 cases (3/6). In addition, the oversized SG was > 20% in 3 cases (3 of 9 cases: > 20% oversized SG). As a result, SG patency was confirmed after 1, 3, 6, and 12 months in 78.6%, 78.6%, 78.6%, and 56.1% of the patients, respectively (Fig. 2). Two cases underwent dual platelet therapy, 3 cases were administered aspirin only, and 1 case had already discontinued the anticoagulation therapy at that time. Two cases, which were in the same patient, experienced SG occlusion 7 days following the procedure with abscesses surrounding the SG (Fig. 1c). In addition, another case had sepsis when the occlusion had occurred. The SG occlusions were not treated, as all the patients were asymptomatic, and no relevant data of ischemic complications were detected. Patients with infection and target artery diameters ≦ 4 mm had significantly higher risks of SG occlusion on the log-rank test (*P* < 0.01 and *P* = 0.01, respectively), while those with > 20% oversized SG were close to significance (*P* = 0.06). On the other hand, anticoagulation therapy did not significantly influence the patency of SGs (*P* = 0.38).

## Discussion

In the present study, EVT with Viabahn SGs was performed in 14 patients with 15 AIBs. In all patients, technical and clinical success was obtained without peripheral organ ischemia. In addition, there were no peri-procedural complications and mortality. These findings suggest that EVT with Viabahn SGs is effective and safe for visceral AIB. Previous studies reported technical success rates of 67–96% in visceral arteries [14–17]. Pedersoli et al. reported technical success with the first attempted intervention in only 6 out of 9 cases (66.7%) [15], indicating achievement of technical success is not to be considered trivial. It is believed that there are several possible reasons for the higher technical success rate in this study than in previous reports. First, the guiding sheath was advanced to cross the injured arteries before SG deployment, since it might not be possible to advance the SG without the guiding sheath through severely torturous vessels. In addition, there is a potential risk of inducing vascular damage by forced advancement of the SG without the guiding sheath, as Viabahn is mounted coverless on the delivery system. Therefore, it may be necessary to cross the guiding sheath to the injured artery before advancing the SG to deliver it safely and certainly as much as possible. In cases where it was dangerous or impossible to advance the guiding sheath to the distal site of bleeding points, a guiding sheath was placed as close as possible to the bleeding point followed by advancing the SG alone over the bleeding point. Second, selecting an appropriate approach site is important. If the abdominal splanchnic artery branches steeply from the aorta to the caudal side, the brachial approach may be easier than the femoral approach for advancing a guiding sheath into the farther side of the visceral artery. Third, the use of a stiff, supported wire as the guidewire ensures the stability of the delivery system.

The SG diameter was 110% the size of the injured artery diameter. Saxon et al. performed EVT with Viabahn for femoropopliteal artery disease, and demonstrated that the primary patency for devices oversized by > 20% was significantly lower than that of devices oversized by < 20% [24]. According to this study, it may be better to avoid devices oversized by > 20%. However, since the most important goal in treating injured arteries is hemostasis, avoiding an undersized diameter is crucial. Therefore, SGs with a diameter of 110% that of injured arteries were selected. Appropriate diameter size selection is important to avoid re-bleeding and ELs. In patients with hypovolemic shock, the artery diameter became small because of spasms; therefore, measurement of the injured artery diameter should be performed using the pre-operative CTA findings.

The neck length is also a critical factor to avoid re-bleeding and type 1 ELs. It should be secured at least 20 mm according to the manufacturer’s instructions for the use of Viabahn; however, this may be challenging, especially in visceral arteries. In the present study, only 9.9 mm (range 1–17 mm) of a mean neck length was ensured in the visceral artery cases, including 1 mm of the shortest neck length. Nevertheless, no post-operative type 1 ELs were observed. Although the neck length is based on the anatomical characteristics, such as angulation and configuration of the target vessel and coagulation ability, our results suggest that it is possible to treat injured arteries without type 1 ELs, even if the neck length is within 20 mm, if the diameter size selection is appropriate. However, if infection, such as biliary or pancreatic leakage, exists surrounding the target vessels, there is a higher risk of re-bleeding or ELs due to the spread of inflammation to the vessel walls. In addition, branch arteries were embolized that were less than 2 mm in diameter within 5 mm of the injured artery to avoid type 2 ELs. Unlike aortic SGs, it is difficult to treat type 2 ELs of peripheral SGs. If a type 2 EL is expected to occur within a branch artery, prophylactic embolization of the branch should be considered before SG replacement.

Although the available CTA imaging follow-up data were limited and were obtained from a small number of patients, the post-operative SG patency was 78.6% after 1, 3, and 6 months and 56.1% after 12 months. Although the SG patency at 1, 3, and 6 months was consistent with previous studies that reported 75–100% [14–20], the SG patency at 12 months was worse. Dual antiplatelet therapy is recommended for at least 6 months following femoral artery stenosis/occlusion treatment with Viabahn to avoid SG occlusion [24]. However, there are no studies on anticoagulation therapy following injured artery treatment. Although anticoagulation therapy based on dual antiplatelet therapy was performed for at least 6 months for most cases in this study, SG occlusions occurred in 5 cases within 12 months. Among these cases, 2 cases experienced occlusion after 12 months, and none of the patients were receiving dual antiplatelet therapy at that time. It is difficult to identify the cause of occlusion; however, other factors, such as the location of the injured artery, infection, oversized SG, and smaller vessel diameters, could be responsible. Indeed, all of the SG occlusions in the present study occurred in visceral artery injuries. Furthermore, 3 cases of SG occlusion had infections (abscesses and sepsis). Additionally, SG oversized by > 20% (the 5 mm SG for 4 mm artery diameter) in 3 cases were used. Moreover, 3 injured arteries were 4 mm in diameter in this study. Lim et al. reported that SG patency in the visceral artery cases was 69.6% at 1 year, and patients with target artery diameters of < 4 mm had a significantly higher risk of SG occlusion [16]. Indeed, our study shows that patients with infection and target artery diameters ≦ 4 mm had significantly higher risk of SG occlusion. In addition, those with > 20% oversized SG tend to have a higher risk of SG occlusion. However, anticoagulation therapy did not significantly influence the patency of SGs. In summary, patients with ≦ 4 mm artery diameter with > 20% oversized SG and infection regardless of anticoagulation therapy might have a higher risk of SG occlusion. Fortunately, all the occlusions did not need treatment since there was no evidence of organ ischemia. Organ ischemia did not occur, probably due to the development of collateral circulation before occlusion formation.

The limitations of the present study include its retrospective nature, single-arm design, and limited imaging follow-up data available for only a small number of patients.

In conclusion, EVT with Viabahn SGs for AIB at the visceral arteries was safe and effective with a low rate of peri-procedural complications and mortality. Additionally, although fixation at least 20 mm from the neck length is recommended, there were no type 1 ELs and re-bleeding even when the neck length was within 20 mm. At midterm, available imaging data showed good SG patency after 1, 3, and 6 months; however, SG occlusions without ischemia of peripheral organs often occurred following 12 months. Long-term follow-up and a larger sample size are needed for further treatment evaluation.

## Abbreviations

AIBs: Arterial injuries and bleedings
CA: Celiac artery
CHA: Common hepatic artery
CTA: Computed tomography angiography
ELs: Endoleaks
EVT: Endovascular treatment
GDA: Gastroduodenal artery
PHA: Proper hepatic artery
RHA: Right hepatic artery
SA: Splenic artery
SG: Stent-graft
SMA: Superior mesenteric artery

## References

[1] Schwartz RA, Teitelbaum GP, Katz MD, Pentecost MJ (1993). Effectiveness of transcatheter embolization in the control of hepatic vascular injuries. J Vasc Interv Radiol.

[2] Hagiwara A, Yukioka T, Ohta S, Tokunaga T, Ohta S, Matsuda H (1997). Nonsurgical management of patients with blunt hepatic injury: efficacy of transcatheter arterial embolization. AJR Am J Roentgenol.

[3] Panetta T, Sclafani SJ, Goldstein AS, Phillips TF (1985). Percutaneous transcatheter embolization for arterial trauma. J Vasc Surg.

[4] Aksoy M, Taviloglu K, Yanar H, Poyanli A, Ertekin C, Rozanes I (2005). Percutaneous transcatheter embolization in arterial injuries of the lower limbs. Acta Radiol.

[5] Phadke RV, Sawlani V, Rastogi H, Kumar S, Roy S, Baijal SS (1997). Iatrogenic renal vascular injuries and their radiological management. Clin Radiol.

[6] Ueda T, Murata S, Yamamoto A, Tamai J, Kobayashi Y, Hiranuma C (2015). Endovascular treatment of post-laparoscopic pancreatectomy splenic arteriovenous fistula with splenic vein aneurysm. World J Gastroenterol.

[7] Parildar M, Oran I, Memis A (2003). Embolization of visceral pseudoaneurysms with platinum coils and N-butyl cyanoacrylate. Abdom Imaging.

[8] Mavili E, Donmez H, Ozcan N, Akcali Y (2007). Endovascular treatment of lower limb penetrating arterial traumas. Cardiovasc Intervent Radiol.

[9] Song HH, Won YD, Kim YJ (2010). Transcatheter N-butyl cyanoacrylate embolization of pseudoaneurysms. J Vasc Interv Radiol.

[10] Tulsyan N, Kashyap VS, Greenberg RK, Sarac TP, Clair DG, Pierce G (2007). The endovascular management of visceral artery aneurysms and pseudoaneurysms. J Vasc Surg.

[11] Venturini M, Marra P, Colombo M, Alparone M, Agostini G, Bertoglio L (2017). Endovascular treatment of visceral artery aneurysms and pseudoaneurysms in 100 patients: covered stenting vs transcatheter embolization. J Endovasc Ther.

[12] Ueda T, Tajima H, Murata S, Takagi R, Yokota H, Kumita SI (2017). Chopstick injury: successful stent-graft therapy for traumatic left subclavian artery aneurysm. J Nippon Med Sch.

[13] Shrikhande GV, Khan SZ, Gallagher K, Morrissey NJ (2011). Endovascular management of superior mesenteric artery pseudoaneurysm. J Vasc Surg.

[14] Venturini M, Marra P, Colombo M, Panzeri M, Gusmini S, Sallemi C (2018). Endovascular repair of 40 visceral artery aneurysms and pseudoaneurysms with the Viabahn stent-graft: technical aspects, clinical outcome and mid-term patency. Cardiovasc Intervent Radiol.

[15] Pedersoli F, Isfort P, Keil S, Goerg F, Zimmermann M, Liebl M (2016). Stentgraft implantation for the treatment of postoperative hepatic artery pseudoaneurysm. Cardiovasc Intervent Radiol.

[16] Lim SJ, Park KB, Hyun DH, Do YS, Park HS, Shin SW (2014). Stent graft placement for postsurgical hemorrhage from the hepatic artery: clinical outcome and CT findings. J Vasc Interv Radiol.

[17] Boufi M, Belmir H, Hartung O, Ramis O, Beyer L, Alimi YS (2011). Emergency stent graft implantation for ruptured visceral artery pseudoaneurysm. J Vasc Surg.

[18] DuBose JJ, Rajani R, Gilani R, Arthurs ZA, Morrison JJ, Clouse WD, Endovascular Skills for Trauma and Resuscitative Surgery Working Group (2012). Endovascular management of axillo-subclavian arterial injury: a review of published experience. Injury.

[19] Kufner S, Cassese S, Groha P (2015). Covered stents for endovascular repair of iatrogenic injuries of iliac and femoral arteries. Cardiovasc Revasc Med.

[20] De Backer O, Arnous S, Sandholt B, Brooks M, Biasco L, Franzen O (2015). Safety and efficacy of using the Viabahn endoprosthesis for percutaneous treatment of vascular access complications after transfemoral aortic valve implantation. Am J Cardiol.

[21] Oliveira NFG, BastosGonçalves FM, Van Rijn MJ, de Ruiter Q, Hoeks S, de Vries JP (2017). Standard endovascular aneurysm repair in patients with wide infrarenal aneurysm necks is associated with increased risk of adverse events. J Vasc Surg.

[22] Leurs LJ, Kievit J, Dagnelie PC, Nelemans PJ, Buth J, EUROSTAR Collaborators (2006). Influence of infrarenal neck length on outcome of endovascular abdominal aortic aneurysm repair. J Endovasc Ther.

[23] Khalilzadeh O, Baerlocher MO, Shyn PB, Connolly BL, Devane AM, Morris CS (2017). Proposal of a new adverse event classification by the society of interventional radiology standards of practice committee. J Vasc Interv Radiol.

[24] Saxon RR, Chervu A, Jones PA, Bajwa TK, Gable DR, Soukas PA (2013). Heparin-bonded, expanded polytetrafluoroethylene-lined stent graft in the treatment of femoropopliteal artery disease: 1-year results of the VIPER (Viabahn Endoprosthesis with Heparin Bioactive Surface in the Treatment of Superficial Femoral Artery Obstructive Disease) trial. J Vasc Interv Radiol.

